# Successful management of an intraoperative electric burn broken ureteral stent: A case report

**DOI:** 10.1016/j.amsu.2022.104849

**Published:** 2022-11-06

**Authors:** Lijun Zhan, Ying Zhan

**Affiliations:** a Department of Urology, Wuhan Tongji Aerospace Hospital, Xinzhou District, Wuhan, Hubei, China; b Department of Urology, Tongji Hospital, Tongji Medical College, Huazhong University of Science and Technology, Wuhan, Hubei, China

**Keywords:** Case report, Double-J-stents, Electrical burn broke stent, Ureteroscopy, KUB, kidney, ureter, and bladder, URS, ureteroscopy, CT, computed tomography

## Abstract

In the treatment of some gynecological diseases, ureteral stents (double J stents) have been generally utilized in the prevention of ureteral injury. Nevertheless, if the ureteral stent is retained as a protective reminder during gynecological surgery, severe ureteral injury can be avoided. Hence, it is very essential to be familiar with the anatomy of ureter in gynecological surgery to prevent complications and morbidity.

We demonstrate a case of a 49-year-old woman who presented with an electric burn broken ureteral stent in the gynecological surgery, but the ureter is only burned but not broken. This resulted in no abnormality being found during surgery. So, ureteroscopy is necessary to extract the ureteral stent and the patient is inserted with another new ureteral stent to repair the ureteral injury.

The electric burn broken ureteral stent is difficult for discovering during the operation. And ureteroscopy is very crucial for the special situation of the ureteral injury.

## Highlights

We present a case of a 49-year-old woman who presented with an electric burn broken ureteral stent in the gynecological surgery, but the ureter is only burned but not broken. This is a very rare case.

• This is a case of electrical burn of ureter during an operation that resulted in the rupture of the D-J protected before surgery.
• The treatment of this injury is simple but difficult to determine preoperatively, which raises a revelation.
• This also provides a reminder that ureteral stent is not completely reliable to protect the ureter, and it still needs to be improved by the surgeon.

## 1 Introduction and importance

The double-J ureteral stent or pigtail stent has turned into a significant tool for ureteral protection in gynecological surgery, especially in some complex gynecological tumor therapy. In the process of gynecological treatment, urologists usually were demanded to give some gynecological patients to be inserted with ureteral stents. When protected stents are encountered, treatment depends on the severity of the injury and ureteral injury, especially the type of injury.

In this study, we have stated a single-session ureteroscopic removal of an electric burn broken ureteral stent for protecting the ureter of the gynecological patient in gynecological surgery. Due to the ureter mucosa burns, so the indwelling double J tube again, after pulling double J stent for three months, follow-up visit medical service found no right ureter stricture and right hydronephrosis.

This case report adheres to the SCARE criteria [1] and highlights the importance of understanding the periureteral anatomy which can help prevent the intraoperative ureteral injury.

## 2 Case presentation

A 49-year-old woman showed with a history of complex gynecological tumor surgery and a protective double-J stent before the operation. However, the double-J ureteral stent was broken with the use of an ultrasound knife or bipolar coagulation unwittingly in the operation. After the operation, the patient had drainage fluid from the abdomen for more than 300ml every day. And she presented with a history of frequency, and intermittent hematuria after the operation.

**Fig. 1:**
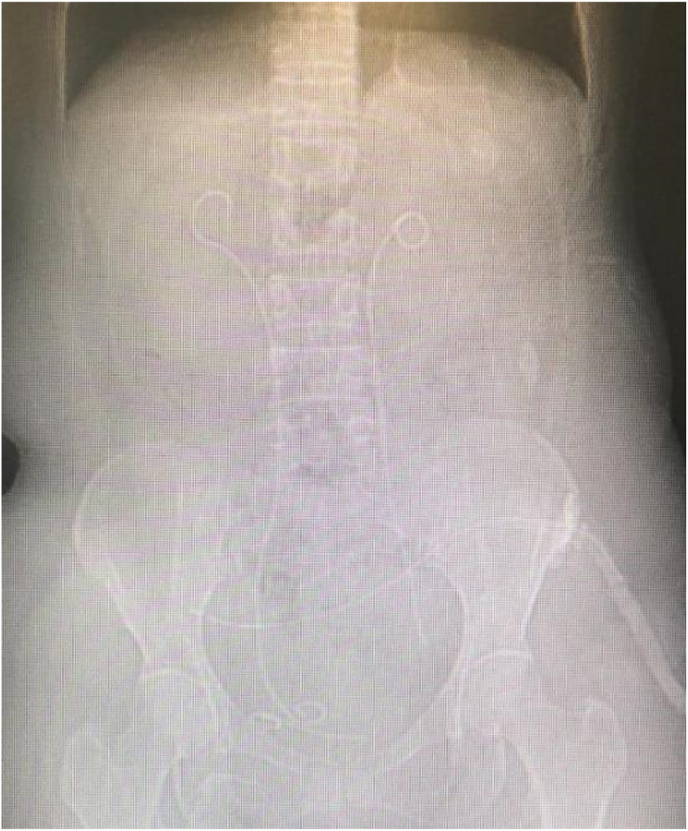
Plain KUB showed left broken Double-J. KUB, kidney, ureter, and bladder radiograph.

The physical examination on admission was not obvious except for slight abdominal tenderness and previous surgical scars. Her hematology and biochemistry were also inconclusive. Urinalysis showed 10–15 pus cells with 15-30RBCs, whereas the urine culture was negative for growth. After examination, the patient was healthy with stable vital signs, pelvic drainage tube was placed during laparoscopic hysterectomy, and other general examinations were normal. Kidney ureter bladder (KUB) x-ray and the computed tomography (CT) showed the broken distal coil of the Double-J stent inside the urinary bladder (Fig. 1).

### 2.1 Intervention

**Fig. 2:**
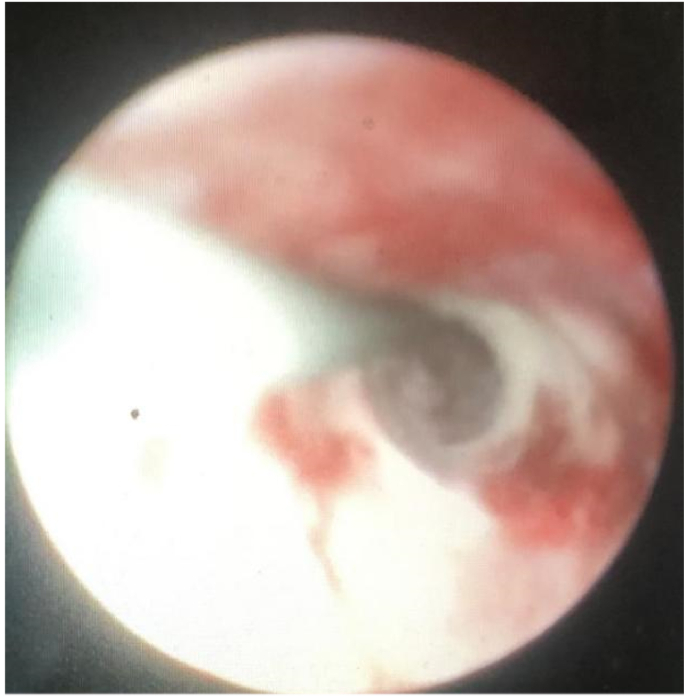
(a and b) ureteroscopic view showing the broken piece of the Double-J and continuous ureter.

**Fig. 3:**
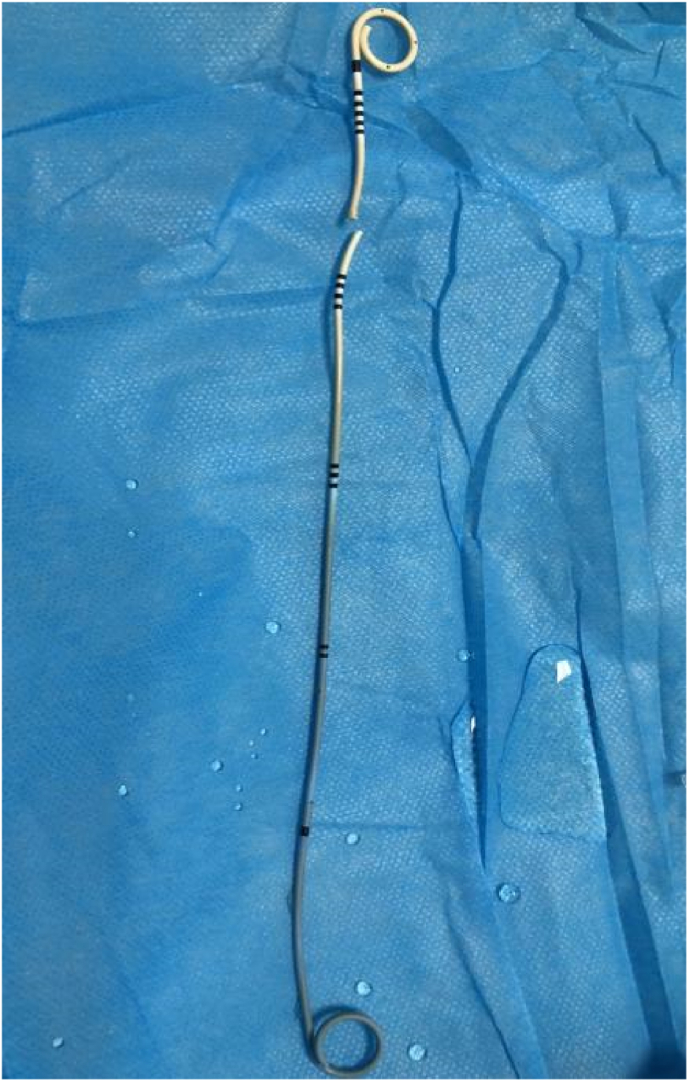
The removed Double-J stent showed a broken piece and the rest of Double-J.

The broken distal coil of Double-J (Fig. 2) extracted the distal coil (which had been broken and separated from the other part of the Double-J stent) was removed alone (Fig. 3). A ureteroscopy (URS) examination revealed a broken end of the ureteral stent tube in the right ureter and a small broken end of the ureteral stent in the bladder. At the same time, burns were found in the ureteral mucosa, belonging to mild burns. The patient was satisfied with the minimally invasive procedure of rein dwelling the ureteral stent. Three months later, the patient underwent cystoscopic removal of the new double J stent without any urinary discomfort.

## 3 Clinical discussion

Ureteral stents (Double-J stents) have been usually used in gynecological surgery to prevent or protect the ureters. Despite technical improvements in stent design and structure, important complications are still reported. Early complications such as fever, vesicle irritative symptoms, and pain are less troublesome than late complications such as fragmentation and/or encrustation of the stent, migration, and infection that can lead to severe morbidity [2,3].

However, broken ureteral stents could lead to numerous complications such as stent migration, stent occlusion, breakage, encrustation, and stone formation [4]. Gynecologists may damage the ureter with an ultrasonic knife in the process of surgery, resulting in the rupture of the double J tube, which causes urine extraversion here, and the drainage fluid in the pelvic drainage tube is more. The ureter was not severed during the procedure, so the gynecologist assumed that the ureter protected by an endourethral stent would be fine during the procedure. But the double J tube has broken off.

The exact reason for electric burn broken ureteral stent is unclear and In this case, that ureter is not severed. It is considered that the heat energy of the ultrasonic knife gathers on the D-J stent, resulting in its separation. In this case, rarely encountered in our clinical work, there are two reasons for this, one is the gynecologists' familiarity with the anatomical structure of the tissue around the ureter; second, the material quality of double J stent is easy to break off for heat aggregation.

In this case, the broken distal coil of Double-J was placed into the bladder and the broken end of the ureteral stent can not be seen at the right ureteral orifice. After entering the right ureter with ureteroscopy, the D-J end was found, but the ureter was not completely severed, only the urethral mucosa was burned. This phenomenon is indeed rare and has not been found in other literature.

The best treatment is the prevention of the intraoperative injury. To avoid the annoyance of the broken double-J stent, some surgeons suggest that gynecologists should be familiar with the anatomy of the ureter and surrounding tissues during surgery. It is pivotal to avoid clamping or ultrasonic knife directly acting on the ureter or adjacent tissues to avoid such situations.

## 4 Conclusion

The electric burn broken ureteral stent is difficult for discovering during the operation. And ureteroscopy is very important for the special situation of the ureteral injury. Meanwhile, it is essential to elucidate the prevention of protective stents in detail for reducing the dependency on gynecologists. In my opinion, a gynecologist's understanding of the anatomy of the ureter and surrounding tissue is the key to avoid ureteral injury.

## Ethical approval

This study was approved by Ethics committee of Wuhan Tongji aerospace hospital.

## Sources of funding

None to declare.

## Author contribution

LJZ wrote the manuscript.

YZ checked all data and revised the manuscript. All authors have read and approved the manuscript.

## Research registration number

1. Name of the registry:
2. Unique Identifying number or registration ID:
3. Hyperlink to your specific registration (must be publicly accessible and will be checked):

## Guarantor

Lijun Zhan^1^, Ying Zhan^2^

^1^ Department of Urology, Wuhan Tongji Aerospace Hospital, Xinzhou District, Wuhan, Hubei, China

^2^ Department of Urology, Tongji Hospital, Tongji Medical College, Huazhong University of Science and Technology, Wuhan, Hubei, China

## Consent

Written informed consent was obtained from the patient for publication of this case report and any accompanying images.We keep the ‘Consent form’ for request anytime.

## Declaration of competing interest

None to declare.

## Abbreviations

kidney, ureter, and bladder=KUB; ureteroscopy=URS; Computed tomography = CT.
